# Dizziness and Convergence Insufficiency in Children: Screening and Management

**DOI:** 10.3389/fnint.2019.00025

**Published:** 2019-07-10

**Authors:** Sylvette R. Wiener-Vacher, Sidney I. Wiener, Layla Ajrezo, Rima Obeid, Damir Mohamed, Priscilla Boizeau, Corinne Alberti, Maria Pia Bucci

**Affiliations:** ^1^Pediatric Balance Evaluation Center (EFEE), ENT Department, AP-HP, Hôpital Robert Debré, Université de Paris, Paris, France; ^2^Hopital Robert Debré, UMR1141 INSERM-Université de Paris, Paris, France; ^3^Center for Interdisciplinary Research in Biology (CIRB), College de France, CNRS, INSERM, PSL Research University, Paris, France; ^4^Clinical Epidemiology Unit, AP-HP, Hôpital Robert Debré, Paris, France; ^5^UMR-S 1123, CIC-EC 1426, INSERM, Université de Paris, Paris, France

**Keywords:** vertigo, pediatric, vergence disorders, saccades, orthoptic training, video screen usage

## Abstract

**Objective:**

In children screened for dizziness with vergence disorders, we tested short and long term efficacy of orthoptic vergence training (OVT) and instructions to reduce screen usage.

**Methods:**

Prospective study: Of the 179 children referred for vertigo or dizziness (over 3 years) with ophthalmological disorder as the only problem after complete oto-neuro-vestibular testing, 69 presented vergence insufficiency, and 49 accepted to participate in this study. 109 healthy children served as controls. All subjects had classic orthoptic evaluation and video binocular movement recordings during various oculomotor tasks. Patients were evaluated before OVT (M0), 3 months after the end of OVT (M3) and 9 months after the end of OVT (M9). Statistics compared orthoptic and oculomotor parameters between patients and controls over time with one-way ANCOVA, and mixed models, controlling for age and gender.

**Results:**

Patients reported vertigo that was usually rotatory, lasting <15 min, associated with or alternating with headache (50%). Their exposure to small video screens and TV was intensive (∼3.6 h per day). At M0, all orthoptic and oculomotor parameters were statistically different in patients relative to controls (*p* < 0.0001) except for divergence. At M3, vertigo symptoms had disappeared in all of the patients, and all eye movement parameters improved significantly (*p* < 0.0001). At M9, this improvement remained stable or continued.

**Conclusion:**

Vergence disorders (assessed by abnormal orthoptic and oculomotor parameters) can generate symptoms of dizziness in children. Orthoptic treatment and instruction to reduce screen usage has a significant and long term effect on vertigo symptoms as well as oculomotor performances. Dizzy children should be screened for vergence disorders.

**WHAT THIS STUDY ADDS:**

Dizziness in children can be associated exclusively with insufficient convergence. Orthoptic training and instructions to reduce screen exposure made dizziness symptoms disappear and improved all eye movement parameters for 6 months. Vergence disorders should be screened for in dizzy children.

## Introduction

It is well known that ophthalmological disorders can induce headaches but most general practitioners, and many specialists, still do not recognize them as a cause of dizziness. We first published evidence that dizziness in children could result from vergence insufficiency VI and dizziness improves when the ophthalmological disorder is treated by orthoptic training, correction of a refractive problem with glasses, or both (Anoh-Tanon et al., 2000). A recent report on a cohort of 1037 vertiginous children found 15% with an ophthalmological disorder as the only cause of dizziness (Wiener-Vacher et al., 2018).

Vertigo (false perceptions of movement of oneself or the environment) and dizziness (sensation of imbalance) may originate at various levels of the central nervous system, where multisensory inputs (including vestibular, somesthetic, proprioceptive, and visual inputs) are integrated (Brandt, 2003; Rine and Braswell, 2003; Braswell and Rine, 2006; Wiener-Vacher et al., 2012; Leigh and Zee, 2015). During rapid head movements, vestibular input triggers oculomotor responses to maintain stable gaze. When the head is fixed, visual inputs trigger a combination of several oculomotor responses: saccades (where gaze jumps from one target to another), smooth pursuit (where gaze follows a slowly moving target), optokinetic responses (where eyes follow the scrolling of the peripheral landscape during movement), and vergence (where both eyes converge on targets getting closer or further away). Dysfunction of these systems can lead to blurry vision, dizziness, vertigo, and headaches particularly after activities requiring intense attention, and convergence during long periods of time (such as reading, playing videogames or looking at mobile telephone screens). Vergence dysfunction can also impair learning activities such as writing or reading (Gaertner et al., 2013; Lions et al., 2013).

In our clinic specialized for diagnosing and treating dizzy children, we observed over the past 5 years an increase of the prevalence of oculomotor disorders as the only cause of the dizziness (10–15%) (Wiener-Vacher et al., 2018). We suggest that this increase of symptomatic vergence disorders may be due to the growing use of electronic devices with small video screens that are very demanding for convergence and diverse controlled saccades during long periods of time. Impacts of computer use on vision have been described by Rosenfield (2016) in adults as “Computer vision syndrome (a.k.a. digital eye strain)” including vergence disorders as well as vertigo. To our knowledge, there are no reports on the effects of video screen exposure on oculomotor disorders in children.

The effects of orthoptic training on oculomotor performance remain controversial. Some publications report that orthoptic training is effective for oculomotor problems such as VI (Van Leeuwen et al., 1999; Bucci et al., 2004; Alvarez et al., 2010; Von Noorden and Campos, 2012) but others claim that this effect is ephemeral (less than a month) (Rawstron et al., 2005). Nevertheless there is evidence that vergence training can modify saccades and vergence parameters in healthy adult subjects (Jainta et al., 2011).

This study shows that dizziness in children can be related to VI (particularly convergence) and occurs in patients with prolonged daily screen exposure. OVT and instructions to reduce screen exposure in these patients has a positive effect on dizziness symptoms as well as correcting oculomotor disorders. Furthermore it shows for the first time that these improvements persist for at least 6 months in these children.

### Patient Group

Forty-nine children (25 boys, 24 girls, 9–13 years old) with VI participated in the study (from 179 children over 3 years diagnosed with ophthalmological problems of which 69 were VI as the only cause of their dizziness). The selection criteria were:

- No history of vestibular, neurological or psychiatric pathology.
- Normal clinical oto-neuro-vestibular examination as described below.
- Absence or minor refraction anomalies (within the range of −1 to +1) with cycloplegic refractometry.
- VI confirmed with ORTE.

Patients were tested before the OVT (M0), 3 months after OVT ended (M3) and 9 months after the end of OVT (M9).

### Control Group

In order to establish normal reference values for the ORTE and the OCME, 109 healthy children (boy/girl ratio = 0.98, 6–17 years old) were recruited from hospital employees’ and patients’ families. All had normal clinical oto-neuro-vestibular evaluations and normal visual acuity.

For ethical reasons we couldn’t test the effect of the OVT on healthy children since they had no complaints and no VI. We evaluated the impact of OVT at M3 and M9 on patients by comparing their oculomotor parameters values to their values at M0 and to those of controls.

Data concerning the symptoms and time of screen exposure for both groups were obtained by asking children and parents.

### Oto-Neuro-Vestibular Evaluation

In both groups, a clinical examination was performed including otologic assessment (otoscopy and acoumetry), neurological and vestibular testing (HIT head impulse test, VOR with videoscopy). Patients had a complete battery of tests to exclude those with any vestibular disorders. This included vestibular canal testing (bithermal caloric test, rotatory chair test, Video Head Impulse Test) and vestibular otolith testing (cervical vestibular evoked myogenic potentials) (Wiener-Vacher et al., 2012).

### Visual Acuity Evaluation

Controls and patients were screened for normal visual acuity in each eye (≥20/20) for far and near viewing. The Parinaud test used for near vision and the Monoyer scale for far vision (Von Noorden and Campos, 2012). Normal refraction for patients was confirmed with cycloplegia between +1 and −1 (with a refractometer after applying drops of 0.5% cyclopentolate, *Skiacol*^®^ to the eyes).

### Initial ORTE

**FIGURE 1:**
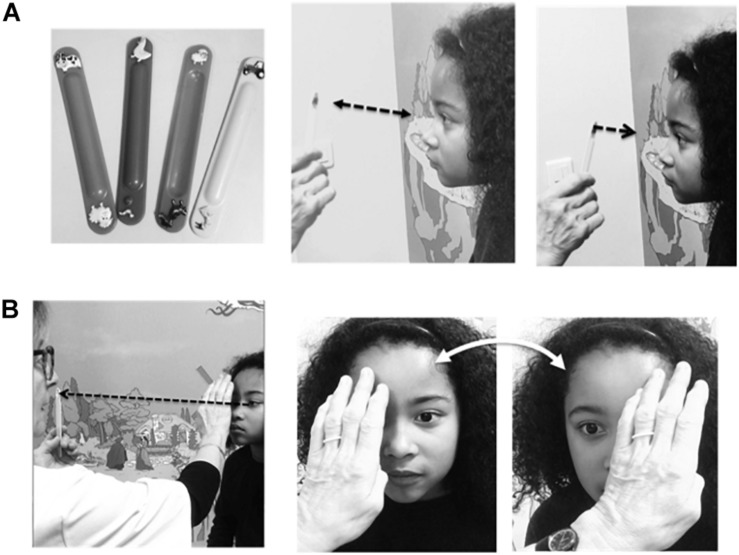
Clinical screening for vergence insufficiency. This test includes 2 steps: **(A)** Looking for an abnormally long NPC during convergence (normal value ≤ 7 cm). (Left) Small targets were child-friendly stickers pasted onto tongue depressors. (Middle) The subject is instructed to fixate on the target as it was moved away from and toward the nose. (Right) As the target approaches the nose, the child must indicate when the target appears double and/or the clinician detects the distance, where smooth binocular eye movements cease and convergence breaks, with one eye ceasing fixation. This corresponds to the NPC. **(B)** Testing for misalignment of the eyes (heterophoria). (Right) Each eye is covered and uncovered by the clinician’s hand while the child fixates on a far target (dashed line and arrow in photo on left). If the eyes remain stable there is no misalignment. If one eye moves when uncovered this indicates a heterophoria.

VI was clinically screened for in both groups by the physician via a simple screening test for weak or asymmetrical eye convergence movements and eye misalignment (see Figure 1 and Supplementary Videos S1, S2). An optometrist assessed vergence performances with a classic test protocol including:

- Measure of near point of convergence (NPC): as distance between nose and target when the target moved toward the nose is first seen as double, or convergence is disrupted (Alvarez et al., 2010; Ajrezo et al., 2016).
- Eye covered-uncovered test: one eye is successively covered and uncovered while the subject gazes at targets at 5 m and 30 cm. If the eyes stay stable when uncovered this excludes a latent eye misalignment corrected by binocular fixation (heterophoria, i.e., latent deviation of a covered eye when the other is not covered); this is not a VI (Cooper, 2011).
- Measurement of the fusion amplitude for divergence and convergence using a Berens prism bar (Cooper, 2011) with targets at 5 m and 30 cm.
- Measurement of the stereoscopic depth discrimination using the TNO (Random dot test, Netherlands Organization for Applied Scientific Research).

The OVT prescribed for all patients included a total of 12 sessions occurring twice a week.

The main goal of OVT was to improve binocular vision for all eye movements at near and far distances. All orthoptists asked patients to perform the same exercises during each session: ocular saccades and pursuits at near distance as well as divergence and convergence at both far and near distances with several instruments: Berens prism bar, synoptophore, and stereograms.

The patients were encouraged to make efforts to increase vergence amplitudes and reach normal values, then to repeat all exercises to make the responses automatic and effortless (Cooper, 2011).

Patients and their families were also encouraged to reduce videoscreen exposure daily. However, it was not possible to reliably track adherence to this recommendation.

### Oculomotor Evaluation

Eye movements were recorded from each eye independently with the Mobile EyeBrain Tracker (Mobile EBT^®^, SuriCog) at a recording frequency of 300 Hz and 0.25° precision. The program permits separate analyses of the different components of the eye movements (saccadic, convergent, and divergent).

**FIGURE 2:**
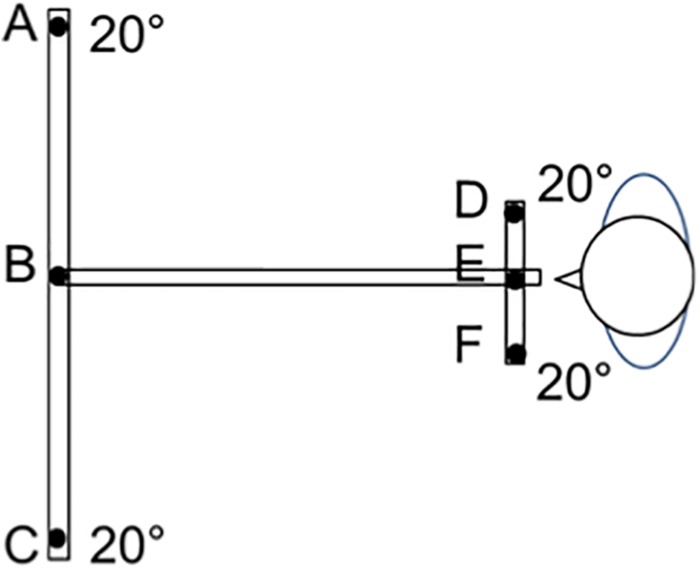
The LED target set up (overhead view). LEDs were presented as targets on a board placed at the child’s eye level. The subject was seated in a dark room, with the head stabilized via a headrest supporting the forehead and chin. Calibration was made during binocular viewing at the beginning of the session (Lions et al., 2013). The child was instructed to look at targets randomly presented at 20° from the midline either at 150 cm (a distance not requiring vergence) or at 20 cm (where convergence is continuously required). Eye movements between adjacent distal targets (between LEDs B and A or B and C) involve saccades alone with no convergence. Eye movements between near targets (E and F or E and D) involve saccades alone with constant convergence. Vergence movements (convergence and divergence alone) were performed for targets presented along the medial plane (convergence from B to E and divergence from E to B). Combined saccade+vergence movements are involved when the target change of distance and laterality from the midline (saccades+divergence between E and A, or E and C, and saccades+convergence B and D, or B and F).

Six conditions of combinations of saccades and vergence were studied: saccades alone with far or near vision (thus imposing constant vergence), divergence and convergence alone without saccades, combined saccades with convergence, and divergence (Figure 2).

### Statistical Analyses

A one-way ANCOVA with control for age and gender was conducted at each evaluation date (M0, M3, and M9) to test for a statistically significant difference in orthoptic and oculomotor parameters between individual patients and the CG.

Mixed models for longitudinal data (MMLD) adjusted for age and gender evaluated the effect of time after training on orthoptic and oculomotor parameters in the patient group (M0 vs. M3, M0 vs. M6, and M3 vs. M6).

For all multiple comparisons, *p*-values were corrected with a Holm adjustment. All statistical tests were two-tailed and *p* < 0.05 is considered statistically significant. All analyses were done with SAS software version 9.4. All *p* values less than 0.0001 are reported as *p* < 0.0001 rather than exact values.

## Results

### Vertigo Symptoms (Figure 3)

Patients reported vertigo as a rotatory sensation in 80% of the cases (40/49) with a sensation of falling in 48% (24/49). The vertigo was usually brief, lasting less than 15 min but recurrent during the day (71%; 35/49) (ranging from once a day to almost continuous in one case).

**FIGURE 3:**
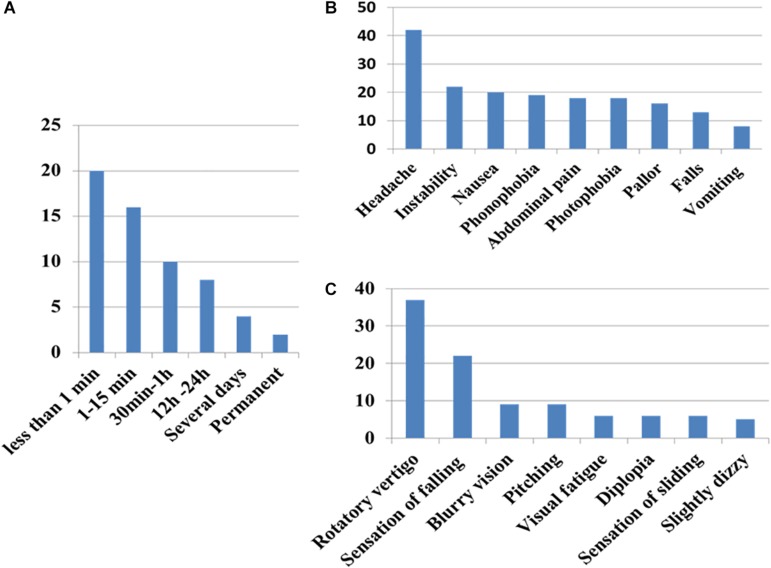
Clinical patterns of symptoms of vergence insufficiency in the patient group **(A)** duration of the sensation **(B)** associated symptoms, and **(C)** sensation perceived. Note that sensation of rotation is never an intense continuous rotatory vertigo as observed in acute vestibular loss.

Headache was reported in 86% (42/49) of the cases, associated with or alternating with vertigo. The pain was frontal in 62%, temporal in 33% or occipital in 16%, pulsatile (56%) or continuous (44%). In 40%, instability, nausea, photo- and phono-phobia, or abdominal pain were experienced during the crises.

Triggering factors were usually stress and ocular fatigue. Children complained at the end of the day (56%) but also in the morning (63%). Symptoms were reported after reading (31%), at school (46%), during or after sport activities (49%), and after using small video screens (31%). The duration of the daily screen exposure (for small screens, mobile phone, video consoles, computer and television) was rather high in the patient group: the mean ± standard deviation was 5.0 **±** 3.3 h per non-school days and 2.3 ± 1.7 h on school days. The weighted average was 3.65 h. The CG reported somewhat less exposure: mean 3.9 **±** 2.1 h per non-school day and 1.6 ± 1.4 h on school days. The weighted average was 2.8 h. The difference between groups did not reach statistical significance, perhaps because of the high variability and the sample size (*p* = 0.054, after correction for age and gender).

Forty-five percentage of the patients reported previous episodes of headache without dizziness or vertigo. Patients’ parents or siblings reported VI in 15% and migraine in 25% of cases.

### M0: All Patients Had Abnormal Orthoptic and Oculomotor Performances Compared to Controls

**FIGURE 4:**
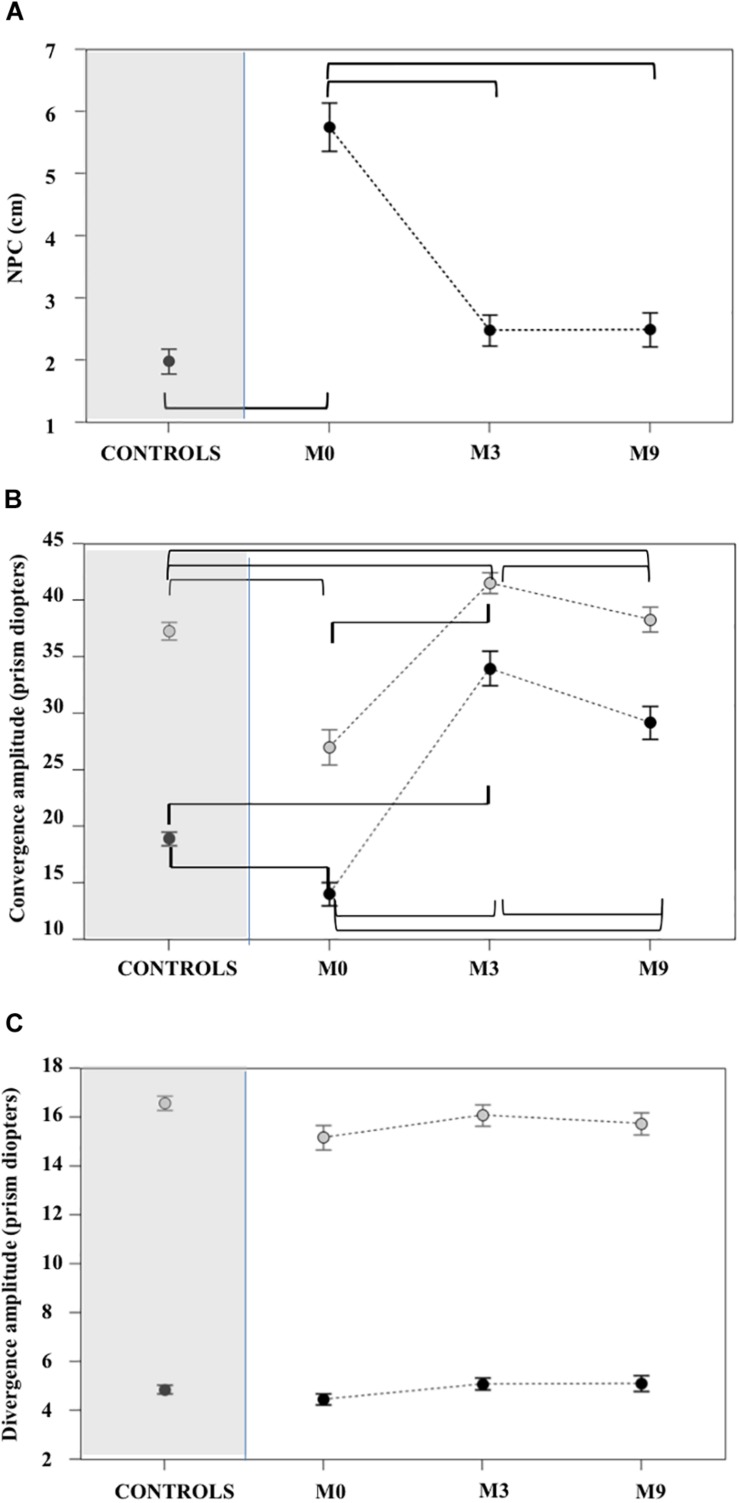
Near point of convergence **(A)**, far and near convergence **(B)** and divergence **(C)** measured with ORTE (mean ± standard deviation). Brackets indicate statistically significant comparisons. At M3 the NPC values for the patient group **(A)** improved relative to M0 and were not significantly different from controls. Amplitudes of near (gray circles) and far (black circles) convergence **(B)** improved significantly in patients at M3 and M9 and these values were greater than controls. Divergence amplitudes **(C)** were not significantly different in patients relative to controls at M0, M3, and M9. For all statistical values see Supplementary Tables S2, S3.

At M0 patients had convergence, but not divergence, insufficiency: the NPC was significantly more distant (Figure 4A and Supplementary Tables S2, S3), amplitudes of convergence at far and near vision were significantly lower in patients than in CG (see Figure 4B and Supplementary Tables S2, S3). In contrast, no patients had divergence amplitudes significantly lower than the controls for near and far vision (Figure 4C and Supplementary Tables S2, S3).

**FIGURE 5:**
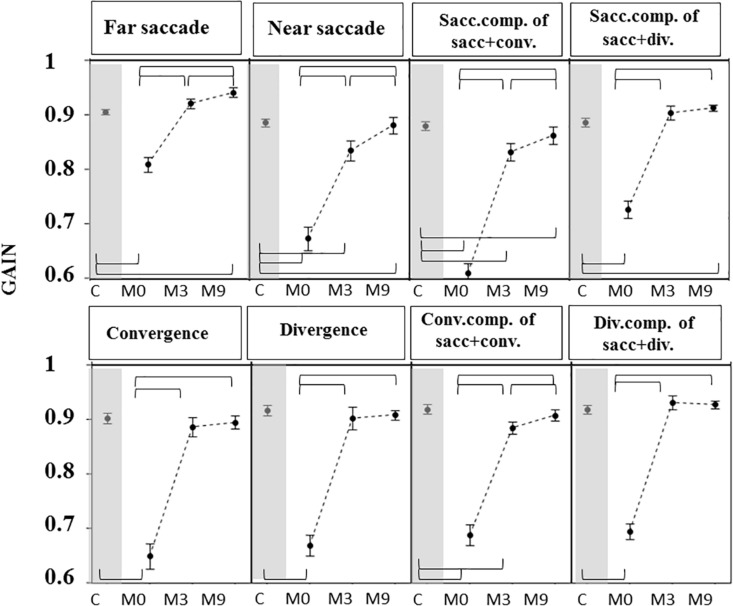
Gain of eye movements measured in OCME for the six test conditions (mean ± standard deviation). For combined saccades+vergence, the saccadic and vergence components are shown individually and labeled: sacc. component, conv. component, and div. component. Statistically significant differences are indicated by braces. Gains at M0 were lower in patients than in controls (C) for all eight conditions. At M3, gains were not significantly different from control values for far saccades, convergence, divergence, and combined saccades with divergence but were still significantly different from controls for near saccades and for both components of saccades combined with convergence. For all statistical values see Supplementary Tables S5, S6.

**FIGURE 6:**
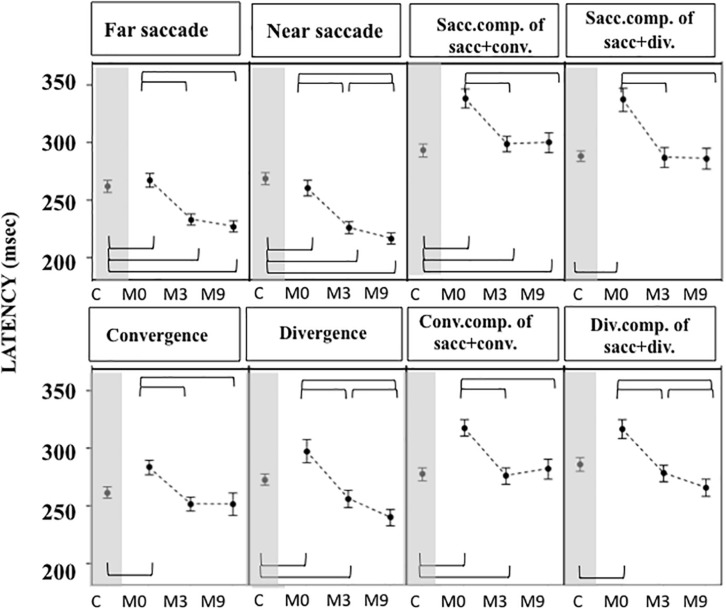
Latencies measured in OCME in the six test conditions (mean and standard deviation). Only statistically significant differences were indicated: top row braces shows differences between M0 vs. M3, M0 vs. M9, and M3 vs. M9 and bottom row shows all differences with controls. Latencies at M0 were significantly longer in patients compared to controls for all conditions tested. At M3 latencies decreased significantly for all conditions after OVT, reaching shorter latencies than controls for near and far saccades. For all statistical values see Supplementary Tables S5, S6.

**FIGURE 7:**
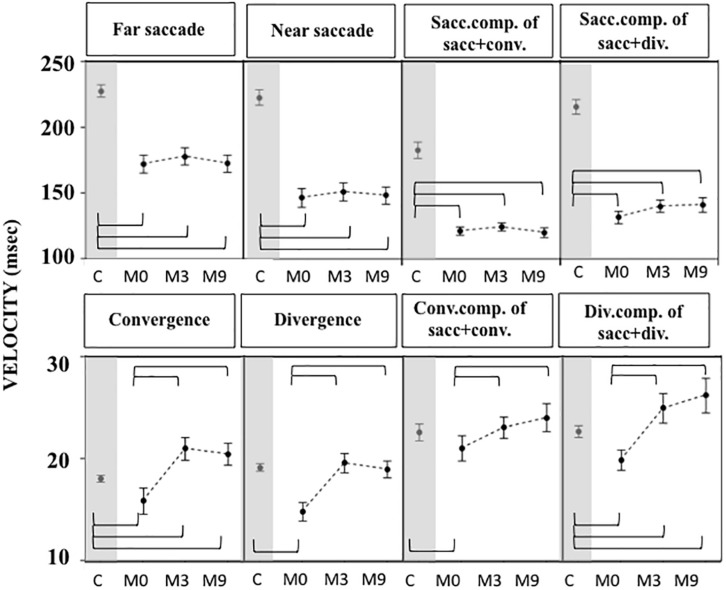
Velocities of eye movements measured in OCME in the eight conditions in controls, and in patients at M0, M3, and M9 (mean and standard deviation). Only statistically significant differences were indicated: top row braces shows differences between M0 vs. M3, M0 vs. M9, and M3 vs. M9 and bottom row shows all differences with controls. Velocities at M0 were significantly lower in patients compared to controls in all conditions except for convergence+saccade, likely because of a compensatory increase of saccade velocity. After training the velocities were still significantly lower in patients than controls for far and near saccades, and for combined saccades with convergence and divergence. In contrast, velocities of vergence movements (alone or combined with saccades) in patients reached values not significantly different from or even higher than controls. For all statistical values see Supplementary Tables S5, S6.

Gains were greater (Figure 5) and latencies (Figure 6) were significantly longer in patients than controls (Supplementary Tables S4, S5) for all conditions tested. Velocities were significantly lower in patients than controls in all conditions (Figure 7 and Supplementary Tables S4, S5).

### M3: Orthoptic and Oculomotor Values Improve After OVT

At M3 the NPC of all patients were not significantly different from controls. Amplitudes of far and near convergence improved significantly and reached values higher than those of controls (Figure 4A and Supplementary Tables S2, S3).

For most of the oculomotor conditions, gain was not significantly different from control values (Figure 5 and Supplementary Tables S4, S5) except for near saccades and combined saccades with convergence. This could be due to remaining weakness of convergence capabilities. Latencies decreased significantly for all conditions, reaching shorter latencies than controls for near and far saccades without vergence (Figure 6 and Supplementary Tables S4, S5). Velocities of vergence alone or combined with saccades were not significantly different from control values (Supplementary Tables S4, S5) or values could be even higher than controls (see Figure 7). Velocities for saccades (far and near) and combined saccades with convergence and divergence remained significantly lower in patients than controls (Supplementary Tables S4, S5).

### M9: Improvement of Orthoptic and Oculomotor Parameters Persists in Patients

Orthoptic parameters in patients remained improved, without significant differences or better when compared to controls at M9 (see Figures 4A–C and Supplementary Tables S1, S2). Oculomotor performances at M9 remained also normal (Figures 5–7 and Supplementary Tables S4, S5).

Gains continued to improve significantly between M3 and M9 for saccades to near and far targets and for saccades+convergence (Figure 5 and Supplementary Tables S4, S5). Latencies continued to decrease significantly between M3 and M9 for saccades to near targets, divergence and divergence+saccades (Figure 6 and Supplementary Tables S4, S5). Velocities did not change significantly from M3 to M9 (Figure 7 and Supplementary Tables S4, S5).

The analysis with mixed models show that improvement of the orthoptic and oculomotor parameters obtained with OVT was statistically significantly maintained over time (Supplementary Tables S3, S6). From M0 to M3 the improvement of all oculomotor parameters was statistically significant (Supplementary Table S6), except for velocities of saccade alone, combined saccades with divergence and convergence.

From M3 to M9, improvements were still statistically significant for some parameters: near and far saccade gains increased and latencies of near saccades decreased significantly (Supplementary Tables S4, S5). The gain of saccades + convergence increased (Figure 4 and Supplementary Tables S4, S5). Latencies of saccades continued to decrease for divergence combined with saccades (see Figure 5 and Supplementary Tables S4, S5).

## Discussion

Children complaining of vertigo or dizziness may have no vestibular pathology, but rather suffer from oculomotor problems including poor saccades and vergence disorders (Anoh-Tanon et al., 2000), in particular convergence insufficiency. Orthoptic treatment led to the disappearance of subjective symptoms of vertigo (Anoh-Tanon et al., 2000) and also improved static and dynamic oculomotor performance. Such improvement persisted and some parameters even progressed further from M3 to M9. At M0, VI patients’ exposure to video screens was found to be intensive (∼3.6 h per day) and this was discouraged.

### Poor Oculomotor Performance in Children With Vertigo

This is the first study recording saccades, vergence and combined saccade-vergence movements before and after orthoptic training in a group of VI children complaining of vertigo but with no vestibular pathology. Before training we measured poor oculomotor performance in these children and found that all made saccades with longer latencies, lower gain and lower velocities than controls. Note that the latency of eye movements includes the preparation time for performing vergence and/or saccadic movements, involving several processes: shift of attention to the visual target, disengagement of oculomotor fixation and computation of the upcoming movement parameters (Fischer, 1987; Seassau and Bucci, 2013). All of these processes are associated with activation of several cortical areas, particularly the parietal cortex and frontal lobe (Leigh and Zee, 2015). In patients with cortical lesions, latencies increase due to cortical dysfunction (Findlay and Walker, 1999). Thus, in the VI children with vertigo, we hypothesize the presence of a minor central dysfunction in the initiation and in the triggering of eye movements.

Our results confirm that the accuracies of saccades and vergence movements are severely impaired in VI children suffering dizziness (Riddell et al., 1990; Bucci et al., 2004; Gaertner et al., 2013). Such poor accuracy could be due to a poor visual localization of the target as a direct consequence of the VI, as suggested previously (Pierrot-Deseilligny et al., 1995; Bucci et al., 2004; Gaertner et al., 2013). Such an impairment in target localization could have an impact on the preparation and the execution of the eye movements, leading to longer latencies, more inaccurate and slower eye movements. These problems could delay learning processes involving saccades and vergence such as reading and writing. Thus screening for VI in children with learning disabilities is of interest.

### Effect of Orthoptic Training

Our study shows that OVT in VI patients can suppress vertigo symptoms and improve saccades and vergence performances (decreasing latencies, improving eye movement precision, and velocity). OVT is widely recommended by clinicians for improving vergence capabilities (Von Noorden and Campos, 2012), but only a very few studies quantitatively showed the effect of OVT on eye movements. Vergence exercises were reported to change vergence movement dynamics in a small population of normal (Von Noorden and Campos, 2012) and VI children (Bucci et al., 2004). Alvarez et al. (2010) observed in adults with VI a correlation between orthoptic training, improvement of vergence dynamics and cerebral changes with fMRI (Alvarez et al., 2010, 2014). Subjects with VI showed a significantly lower activation of cerebellar vermis, frontal and parietal cortex with vergence movements compared to healthy adults with no VI, and this activity improved significantly after a total of 18 h of OVT (Alvarez et al., 2014). These findings suggest that OVT may act at a central level. The continued improvement in oculomotor performances we observed 6 months after the end of the OVT support the hypothesis of central changes. However, this does not exclude a peripheral effect of training on eye muscles.

### Clinical Considerations

Quantitative recordings of eye movements together with clinical orthoptic tests proved useful here for detailed diagnosis and treatment follow-up of dizzy children with no vestibular or neurological disorder. OVT improved their VI and this persisted and even continued to improve after the end of the training.

It is thus important to promote screening of VI in vertiginous children, in particular those with neither vestibular nor neurological disorders, and if vergence disorder is found to prescribe ORTE.

In our pediatric balance evaluation center, vertigo due to vergence disorders is the second more frequent diagnosis after migraine; its prevalence increases every year (from 10% in 2014 to 15% in 2018) (Wiener-Vacher et al., 2018). The American Association of Pediatrics (Swing et al., 2010) recommended limiting children’s exposure to video screens for many reasons. Our results show that symptomatic cases of VI tend to be associated with longer exposure to small video screens than controls. Today many children are exposed to small video screens for long periods of time. The increasing prevalence of symptomatic VI that we observed could be explained by this lifestyle. For health care and prevention, children and particularly those who suffer VI should be advised to reduce video screen exposure. Note, however, that video screen exposure was on average 2.8 h per day year around for the CG while it was 3.6 h per day for the dizzy patients with VI. Thus despite their intensive screen exposure, controls were not dizzy and that would support the hypothesis of a pre-existing latent or minor vergence disorders that were aggravated by the intense exposure in patients.

## Conclusion

Pediatric patients with vertigo and dizziness associated with convergence insufficiency benefit from orthoptic training with a significant improvement of the vertigo symptoms as well as oculomotor performances. These effects persist after the end of OVT and even continue to progress for some parameters. Vergence disorders should be screened for in all dizzy children with normal neurological and vestibular examinations, and then followed by treatment and instructions for reduced video screen exposure.

## Data Availability

The raw data supporting the conclusions of this manuscript will be made available by the authors, without undue reservation, to any qualified researcher.

## Ethics Statement

This investigation adhered to the Declaration of Helsinki principles and was approved by our local Human Experimentation Committee (10801-AOR09078, AFSSAPS B100388-40). Written consent was obtained from the children’s parents after careful review of the experimental procedures with them.

## Author Contributions

SW-V and MPB conceived and designed the study, drafted the initial manuscript, reviewed the manuscript for intellectual content, and finalized the submitted version. SIW performed a statistical analysis, reviewed the manuscript for intellectual content, revised the manuscript, and finalized the submitted version. RO and LA designed the data collection instruments, collected the data, and carried out the initial analyses. CA, DM, and PB developed the data base and carried out the statistical analyses. All authors approved the final manuscript as submitted and agreed to be accountable for all aspects of the work.

## Conflict of Interest Statement

The authors declare that the research was conducted in the absence of any commercial or financial relationships that could be construed as a potential conflict of interest. The handling Editor declared a shared affiliation, though no other collaboration, with authors MBP and SIW.

## Supplementary Material

TABLE S1 Orthoptic parameters for control and patient groups at M0, M3, and M9.

TABLE S2 Mean ANCOVA differences between patient and control groups and 95% confidence interval for orthoptic parameters (multiple comparisons controlling for age and gender). Significant values are highlighted in gray.

TABLE S3 The effect of orthoptic training over time on orthoptic parameters with mixed models for longitudinal data (mean differences with minimum-maximum of the 95% confidence interval). Same format as Supplementary Table S2.

TABLE S4 Mean values of oculomotor parameters (with standard deviations) over all conditions of the saccade/vergence for controls and patient groups at M0, M3, and M9.

TABLE S5 Mean ANCOVA differences mean with minimum-maximum of the 95% confidence intervals in oculomotor parameters between patient and control groups (Multiple comparisons controlling for age and gender). Statistically significant differences are highlighted in gray.

TABLE S6 The effect of orthoptic training over time on oculomotor parameters with mixed models for longitudinal data (mean differences with minimum-maximum of the 95% confidence interval). Statistically significant differences are highlighted in gray.

VIDEO S1 Screening for weak or asymmetrical vergence eye movements and misalignment.

VIDEO S2 Left eye weak convergence and without eye misalignment.

## Abbreviations

CG: control group
M0: initial evaluation before OVT
M3: evaluation 3 months after M0 and the end of OVT
M9: evaluation 9 months after the end of OVT
NPC: near point of convergence
OCME: oculomotor evaluation
ORTE: orthoptic evaluation
OVT: orthoptic vergence training
VI: vergence insufficiency.
