# Supplementary material for: Dizziness and Convergence Insufficiency in Children: Screening and Management
Source: Front Integr Neurosci. 2019 Jul 10;13:25. doi: 10.3389/fnint.2019.00025 (PMC6636600; doi:10.3389/fnint.2019.00025)
Supplement: TABLE S1 — Orthoptic parameters for control and patient groups at M0, M3, and M9. [file Table_1.pdf]

**Orthoptic examination parameters**	**Controls**	**Patients M0**	**Patients M3**	**Patients M9**
NPC (cm)	2.0 (0.2)	5.8 (0.4)	2.5 (0.3)	2.5 (0.3)
Far convergence amplitude (prism diopters)	18.9 (0.6)	14 (1)	34 (1.5)	29.2 (1.4)
Near convergence amplitude (prism diopters)	37.3 (0.8)	27 (1.6)	41.5 (0.9)	38.3 (1.1)
Far divergence amplitude (prism diopters)	4.9 (0.2)	4.5 (0.2)	5.1 (0.2)	5.1 (0.3)
Near divergence amplitude (prism diopters)	16.6 (0.3)	15.2 (0.5)	16.1 (0.4)	15.7 (0.4)

Table 1
ORTE
