# Supplementary material for: Dizziness and Convergence Insufficiency in Children: Screening and Management
Source: Front Integr Neurosci. 2019 Jul 10;13:25. doi: 10.3389/fnint.2019.00025 (PMC6636600; doi:10.3389/fnint.2019.00025)
Supplement: TABLE S2 — Mean ANCOVA differences between patient and control groups and 95% confidence interval for orthoptic parameters (multiple comparisons controlling for age and gender). Significant values are highlighted in gray. [file Table_2.pdf]

Orthoptic examination parameters	M0 : patients vs controls		M3 : patients vs controls		M9 : patients vs controls	
NPC (cm)	3.68 [2.69 ; 4.66] p<.0001	F=79.25	0.42 [-0.38 ; 1.22] p=0.48	F=1.39	0.38 [-0.34 ; 1.11] p=0.3	F=1.08
Far convergence amplitude (prism diopters)	-4.60 [-6.94 ; -2.25] p=0.0002	F=14.77	15.04 [11.63 ; 18.44] p<.0001	F=111.84	10.18 [7.05 ; 13.32] p<.0001	F=52.96
Near convergence amplitude (prism diopters)	-9.43 [-13.24 ; -5.63] p<.0001	F=35.29	5.10 [2.16 ; 8.04] p=0.0003	F=15.08	1.76 [-1.00 ; 4.52] p=0.21	F=1.57
Far divergence amplitude (prism diopters)	-0.42 [-1.20 ; 0.37] p=0.62	F=1.62	0.14 [-0.51 ; 0.79] p=0.68	F=0.17	0.18 [-0.64 ; 0.99] p=1.00	F=0.24
Near divergence amplitude (prism diopters	-0.90 [-2.24 ; 0.44] p=0.33	F=2.61	-0.03 [-1.07 ; 1.02] p=0.96	F=0.00	-0.32 [-1.54 ; 0.90] p=1	F=0.34

Table 2
ORTE
