# Supplementary material for: Dizziness and Convergence Insufficiency in Children: Screening and Management
Source: Front Integr Neurosci. 2019 Jul 10;13:25. doi: 10.3389/fnint.2019.00025 (PMC6636600; doi:10.3389/fnint.2019.00025)
Supplement: TABLE S3 — The effect of orthoptic training over time on orthoptic parameters with mixed models for longitudinal data (mean differences with minimum-maximum of the 95% confidence interval). Same format as Supplementary Table S2. [file Table_3.pdf]

Orthoptic examination parameters	M3 vs M0		M9 vs M0		M9 vs M3	
NPC (cm)	-3.26 [-4.15 ; -2.37]	p<.0001	-3.23 [-4.16 ; -2.30]	p<.0001	0.03 [-0.57 ; 0.63]	p=0.92
far convergence amplitude (prism diopters)	20.04 [15.82 ; 24.26]	p<.0001	15.18 [11.59 ; 18.77]	p<.0001	-4.86 [-7.96 ; -1.76]	p=0.004
Near convergence amplitude (prism diopters)	14.54 [10.39 ; 18.68]	p<.0001	11.19 [7.23 ; 15.15]	p<.0001	-3.34 [-5.11 ; -1.57]	p=0.001
far divergence amplitude (prism diopters)	0.64 [0.01 ; 1.26]	p=0.06	0.62 [-0.02 ; 1.26]	p=0.07	-0.02 [-0.48 ; 0.44]	p=0.94
Near divergence amplitude (prism diopters)	0.92 [-0.13 ; 1.97]	p=0.12	0.53 [-0.68 ; 1.74]	p=0.67	-0.39 [-1.25 ; 0.46]	p=0.37

Table 3
ORTE
