# Supplementary material for: Dizziness and Convergence Insufficiency in Children: Screening and Management
Source: Front Integr Neurosci. 2019 Jul 10;13:25. doi: 10.3389/fnint.2019.00025 (PMC6636600; doi:10.3389/fnint.2019.00025)
Supplement: TABLE S4 — Mean values of oculomotor parameters (with standard deviations) over all conditions of the saccade/vergence for controls and patient groups at M0, M3, and M9. [file Table_4.pdf]

Types of eye movement	Parameters	Controls	Patients M0	Patients M3	Patients M9
Far saccade	Gain	0.91 (0.01)	0.81 (0.01)	0.92 (0.01)	0.94 (0.01)
	Latency (ms)	262.38 (5.20)	267.78 (6.07)	233.39 (4.99)	227.31 (4.77)
	Velocity (°/sec)	227.76 (4.76)	172.29 (7.04)	178.10 (6.54)	172.78 (6.49)
Near saccade	Gain	0.89 (0.01)	0.67 (0.02)	0.83 (0.02)	0.88 (0.02)
	Latency (ms)	269.24 (5.16)	260.56 (6.62)	226.17 (5.11)	216.67 (4.82)
	Velocity (°/sec)	222.86 (6.13)	146.54 (7.22)	151.16 (6.91)	148.32 (6.31)
Convergence	Gain	0.90 (0.01)	0.65 (0.02)	0.89 (0.02)	0.89 (0.01)
	Latency (ms)	261.87 (4.58)	337.92 (10.10)	251.79 (5.93)	251.62 (9.85)
	Velocity (°/sec)	18.04 (0.34)	15.86 (1.28)	21.00 (1.11)	20.47 (1.06)
Divergence	Gain	0.92 (0.01)	0.67 (0.02)	0.90 (0.02)	0.91 (0.01)
	Latency (ms)	272.98 (4.94)	297.80 (10.24)	256.49 (7.55)	240.17 (7.28)
	Velocity (°/sec)	19.14 (0.38)	14.81 (0.91)	19.59 (0.94)	18.97 (0.82)
Saccadic component of combined saccade+convergence	Gain	0.88 (0.01)	0.61 (0.02)	0.83 (0.02)	0.86 (0.02)
	Latency (ms)	293.42 (5.59)	338.80 (8.00)	298.93 (6.95)	300.12 (8.64)
	Velocity (°/sec)	182.85 (6.29)	121.27 (3.28)	124.12 (3.17)	120.08 (3.60)
Saccadic component of combined saccade+divergence	Gain	0.89 (0.01)	0.73 (0.02)	0.90 (0.01)	0.91 (0.01)
	Latency (ms)	288.42 (4.79)	337.92 (10.10)	287.32 (8.92)	286.29 (9.18)
	Velocity (°/sec)	215.80 (5.50)	131.62 (4.79)	140.06 (4.77)	141.25 (5.49)
Convergent component of combined saccade+convergence	Gain	0.92 (0.01)	0.69 (0.02)	0.88 (0.01)	0.91 (0.01)
	Latency (ms)	277.69 (5.57)	317.99 (6.89)	276.29 (7.00)	282.32 (8.70)
	Velocity (°/sec)	22.62 (0.83)	21.04 (1.25)	23.08 (1.05)	24.04 (1.36)
Divergent component of combined saccade+divergence	Gain	0.92 (0.01)	0.69 (0.01)	0.93 (0.01)	0.93 (0.01)
	Latency (ms)	286.17 (5.74)	317.18 (8.21)	278.46 (6.98)	266.00 (7.55)
	Velocity (°/sec)	22.69 (0.57)	19.87 (0.97)	24.98 (1.47)	26.25 (1.71)

Table 4
OCME
