# Supplementary material for: Dizziness and Convergence Insufficiency in Children: Screening and Management
Source: Front Integr Neurosci. 2019 Jul 10;13:25. doi: 10.3389/fnint.2019.00025 (PMC6636600; doi:10.3389/fnint.2019.00025)
Supplement: TABLE S5 — Mean ANCOVA differences mean with minimum-maximum of the 95% confidence intervals in oculomotor parameters between patient and control groups (Multiple comparisons controlling for age and gender). Statistically significant differences are highlighted in gray. [file Table_5.pdf]

Eye movement type	Parameters	M0 : patients vs controls		M3 : patients vs controls		M9 : patients vs controls	
Far saccade	Gain	-0.11 [-0.13 ; -0.08] p<.0001	F=138.12	0.01 [-0.01 ; 0.02] p=0.3	F=1.08	0.03 [0.01 ; 0.05] p=0.002	F=10.59
	Latency (ms)	21.92 [6.98 ; 36.87] p=0.002	F=12.34	-12.85 [-24.90 ; -0.79] p=0.04	F=4.36	-16.81 [-31.17 ; -2.45] p=0.02	F=6.89
	Velocity (°/sec)	-54.35 [-72.30 ; -36.40] p<.0001	F=52.54	-47.13 [-61.71 ; -32.56] p<.0001	F=40.18	-52.85 [-70.03 ; -35.66] p<.0001	F=47.51
Near saccade	Gain	-0.23 [-0.26 ; -0.20] p<.0001	F=322.95	-0.07 [-0.09 ; -0.04] p<.0001	F=30.26	-0.02 [-0.04 ; 0.00] p=0.1	F=2.66
	Latency (ms)	7.98 [-4.50 ; 20.45] p=0.21	F=1.57	-26.81 [-40.85 ; -12.77] p<.0001	F=18.32	-34.17 [-49.62 ; -18.72] p<.0001	F=28.03
	Velocity (°/sec)	-71.62 [-94.07 ; -49.16] p<.0001	F=58.28	-65.91 [-84.16 ; -47.66] p<.0001	F=50.1	-68.23 [-89.63 ; -46.82] p<.0001	F=51.06
Convergence	Gain	-0.27 [-0.31 ; -0.23] p<.0001	F=262.72	-0.03 [-0.07 ; 0.00] p=0.10	F=3.82	-0.03 [-0.05 ; 0.00] p=0.09	F=2.81
	Latency (ms)	36.69 [22.65 ; 50.72] p<.0001	F=39.15	4.56 [-6.81 ; 15.94] p=0.43	F=0.62	7.41 [-7.33 ; 22.15] p=0.52	F=1.27
	Velocity (°/sec)	-2.25 [-3.66 ; -0.83] p=0.002	F=9.66	2.83 [1.22 ; 4.45] p<.0001	F=17.64	2.28 [0.78 ; 3.77] p=0.001	F=11.57
Divergence	Gain	-0.27 [-0.31 ; -0.23] p<.0001	F=302.79	-0.03 [-0.06 ; -0.00] p=0.05	F=4.00	-0.03 [-0.06 ; 0.00] p=0.06	F=4.62
	Latency (ms)	40.59 [24.06 ; 57.13] p<.0001	F=34.53	-1.05 [-13.64 ; 11.54] p=0.87	F=0.03	-15.90 [-30.81 ; -0.99] p=0.03	F=5.71
	Velocity (°/sec)	-4.31 [-5.92 ; -2.70] p<.0001	F=40.99	0.57 [-0.95 ; 2.09] p=0.81	F=0.70	-0.19 [-1.49 ; 1.11] p=0.78	F=0.08
Saccadic component of combined saccade+convergence	Gain	-0.28 [-0.31 ; -0.25] p<.0001	F=530.21	-0.06 [-0.09 ; -0.03] p<.0001	F=25.47	-0.03 [-0.06 ; -0.01] p=0.009	F=6.96
	Latency (ms)	62.00 [44.42 ; 79.58] p<.0001	F=71.29	21.86 [7.64 ; 36.08] p=0.003	F=9.08	25.91 [8.65 ; 43.16] p=0.002	F=11.32
	Velocity (°/sec)	-64.45 [-86.73 ; -42.17] p<.0001	F=47.97	-61.76 [-79.97 ; -43.55] p<.0001	F=44.18	-66.35 [-87.96 ; -44.74] p<.0001	F=47.35
Saccadic component of combined saccade+divergence	Gain	-0.17 [-0.20 ; -0.14] p<.0001	F=164.9	0.01 [-0.01 ; 0.04] p=0.24	F=1.36	0.02 [-0.00 ; 0.05] p=0.13	F=3.37
	Latency (ms)	62.28 [45.80 ; 78.76] p<.0001	F=81.89	10.95 [-2.32 ; 24.23] p=0.11	F=2.61	11.41 [-4.24 ; 27.05] p=0.21	F=2.67
	Velocity (°/sec)	-80.25 [-100.34 ; -60.15] p<.0001	F=91.36	-71.07 [-89.61 ; -52.52] p<.0001	F=73.79	-69.88 [-86.82 ; -52.94] p<.0001	F=65.36
Convergent component of combined convergence+saccade	Gain	-0.24 [-0.27 ; -0.20] p<.0001	F=258.69	-0.04 [-0.07 ; -0.01] p=0.008	F=8.48	-0.02 [-0.04 ; 0.01] p=0.27	F=1.24
	Latency (ms)	55.21 [37.47 ; 72.95] p<.0001	F=55.51	12.87 [-1.95 ; 27.69] p=0.09	F=2.9	21.28 [3.26 ; 39.30] p=0.02	F=7.00
	Velocity (°/sec)	-0.90 [-3.48 ; 1.68] p=0.5	F=0.46	1.23 [-1.65 ; 4.11] p=0.68	F=0.92	2.47 [-0.79 ; 5.72] p=0.21	F=3.3
Divergent component of combined divergence+saccade	Gain	-0.23 [-0.26 ; -0.20] p<.0001	F=351.56	0.01 [-0.02 ; 0.03] p=1.00	F=0.34	0.00 [-0.02 ; 0.02] p=0.86	F=0.03
	Latency (ms)	47.30 [29.32 ; 65.27] p<.0001	F=39.7	8.18 [-8.43 ; 24.78] p=0.54	F=1.22	-1.90 [-17.00 ; 13.20] p=0.81	F=0.06
	Velocity (°/sec)	-2.66 [-4.73 ; -0.58] p=0.009	F=8.26	2.60 [0.59 ; 4.60] p=0.01	F=6.44	3.95 [1.33 ; 6.57] p=0.001	F=13.03

Table 5
OCME
