# Supplementary material for: Dizziness and Convergence Insufficiency in Children: Screening and Management
Source: Front Integr Neurosci. 2019 Jul 10;13:25. doi: 10.3389/fnint.2019.00025 (PMC6636600; doi:10.3389/fnint.2019.00025)
Supplement: TABLE S6 — The effect of orthoptic training over time on oculomotor parameters with mixed models for longitudinal data (mean differences with minimum-maximum of the 95% confidence interval). Statistically significant differences are highlighted in gray. [file Table_6.pdf]

Eye movement type	Prameters	M3 vs M0		M9 vs M0		M9 vs M3	
Far saccade	Gain	0.11 [0.08 ; 0.14]	p<0.0001	0.13 [0.09 ; 0.17]	p<0.0001	0.02 [0.01 ; 0.03]	p=0.0004
	Latency (ms)	-34.39 [-44.83 ; -23.96]	p<0.0001	-39.38 [-50.71 ; -28.05]	p<0.0001	-4.99 [-13.13 ; 3.15]	p=0.24
	Velocity (°/sec)	5.80 [-12.79 ; 24.40]	p=0.99	3.04 [-13.39 ; 19.48]	p=0.72	-2.76 [-9.72 ; 4.19]	p>.99
Near saccade	Gain	0.16 [0.11 ; 0.21]	p<0.0001	0.20 [0.15 ; 0.25]	p<0.0001	0.04 [0.02 ; 0.06]	p=0.0004
	Latency (ms)	34.39 [-47.69 ; -21.08]	p<0.0001	-44.15 [-59.94 ; -28.35]	p<0.0001	-9.76 [-16.68 ; -2.84]	p=0.01
	Velocity (°/sec)	4.61 [-10.57 ; 19.80]	p=0.99	1.53 [-9.54 ; 12.60]	p=0.79	-3.09 [-10.22 ; 4.05]	p=0.92
Convergence	Gain	0.24 [0.18 ; 0.29]	p<0.0001	0.25 [0.19 ; 0.30]	p<0.0001	0.01 [-0.01 ; 0.03]	p=0.39
	Latency (ms)	-31.87 [-39.17 ; -24.56]	p<0.0001	-32.72 [-51.88 ; -13.56]	p=0.001	-0.86 [-15.75 ; 14.04]	p=0.91
	Velocity (°/sec)	5.23 [2.61 ; 7.85]	p<0.0001	4.81 [2.22 ; 7.40]	p=0.0003	-0.41 [-1.65 ; 0.82]	p=0.51
Divergence	Gain	0.23 [0.17 ; 0.30]	p<0.0001	0.24 [0.19 ; 0.29]	p<0.0001	0.01 [-0.02 ; 0.04]	p=0.61
	Latency (ms)	41.31 [-53.58 ; -29.03]	p<0.0001	-58.10 [-75.47 ; -40.73]	p<0.0001	-16.79 [-27.79 ; -5.79]	p=0.004
	Velocity (°/sec)	4.77 [2.79 ; 6.76]	p<0.0001	4.19 [2.25 ; 6.14]	p<0.0001	-0.58 [-2.11 ; 0.95]	p=0.46
Saccadic component of combined saccade+convergence	Gain	0.22 [0.18 ; 0.27]	p<0.0001	0.25 [0.20 ; 0.30]	p<0.0001	0.03 [0.01 ; 0.04]	p=0.01
	Latency (ms)	-39.87 [-54.22 ; -25.52]	p<0.0001	-39.56 [-60.21 ; -18.91]	p<0.0001	0.31 [-13.35 ; 13.97]	p=0.99
	Velocity (°/sec)	2.86 [-3.53 ; 9.24]	p=0.64	-0.27 [-6.88 ; 6.34]	p=0.94	-3.12 [-8.58 ; 2.33]	p=0.53
Saccadic component of combined saccade+divergence	Gain	0.18 [0.13 ; 0.22]	p<0.0001	0.19 [0.15 ; 0.23]	p<0.0001	0.01 [-0.01 ; 0.03]	p=0.3
	Latency (ms)	-50.60 [-66.83 ; -34.37]	p<0.0001	-54.73 [-74.12 ; -35.35]	p<0.0001	-4.13 [-11.43 ; 3.17]	p=0.27
	Velocity (°/sec)	6.97 [-2.35 ; 16.29]	p=0.2	11.37 [-1.62 ; 24.36]	p=0.12	4.40 [-4.58 ; 13.38]	p=0.34
Convergent component of combined saccade+convergence	Gain	0.20 [0.16 ; 0.24]	p<0.0001	0.22 [0.18 ; 0.27]	p<0.0001	0.03 [0.01 ; 0.04]	p<0.0001
	Latency (ms)	-41.70 [-53.07 ; -30.33]	p<0.0001	-35.76 [-55.90 ; -15.62]	p=0.001	5.95 [-9.46 ; 21.35]	p=0.45
	Velocity (°/sec)	2.04 [0.11 ; 3.97]	p=0.04	3.18 [0.25 ; 6.10]	p=0.04	1.13 [-0.78 ; 3.05]	p=0.25
Divergent component of combined saccade+divergence	Gain	0.24 [0.19 ; 0.28]	p<0.0001	0.23 [0.19 ; 0.28]	p<0.0001	-0.00 [-0.02 ; 0.01]	p=0.79
	Latency (ms)	-38.72 [-51.70 ; -25.74]	p<0.0001	-50.25 [-65.38 ; -35.12]	p<0.0001	-11.52 [-20.11 ; -2.94]	p=0.01
	Velocity (°/sec)	5.11 [2.30 ; 7.91]	p=0.0004	6.36 [3.22 ; 9.51]	p<0.0001	1.26 [-0.82 ; 3.33]	p=0.24

Table 6
OCME
